# Hypersialylation in Cancer: Modulation of Inflammation and Therapeutic Opportunities

**DOI:** 10.3390/cancers10060207

**Published:** 2018-06-18

**Authors:** Emily Rodrigues, Matthew S. Macauley

**Affiliations:** 1 Department of Chemistry, University of Alberta, Edmonton, AB T6G 2G2, Canada; er5@ualberta.ca; 2 Department of Medical Microbiology and Immunology, University of Alberta, Edmonton, AB T6G 2G2, Canada

**Keywords:** sialic acid, Siglec, Selectin, inflammation, lectin, glycosylation, tumor-associated macrophage, immunosurveillance

## Abstract

Cell surface glycosylation is dynamic and often changes in response to cellular differentiation under physiological or pathophysiological conditions. Altered glycosylation on cancers cells is gaining attention due its wide-spread occurrence across a variety of cancer types and recent studies that have documented functional roles for aberrant glycosylation in driving cancer progression at various stages. One change in glycosylation that can correlate with cancer stage and disease prognosis is hypersialylation. Increased levels of sialic acid are pervasive in cancer and a growing body of evidence demonstrates how hypersialylation is advantageous to cancer cells, particularly from the perspective of modulating immune cell responses. Sialic acid-binding receptors, such as Siglecs and Selectins, are well-positioned to be exploited by cancer hypersialylation. Evidence is also mounting that Siglecs modulate key immune cell types in the tumor microenvironment, particularly those responsible for maintaining the appropriate inflammatory environment. From these studies have come new and innovative ways to block the effects of hypersialylation by directly reducing sialic acid on cancer cells or blocking interactions between sialic acid and Siglecs or Selectins. Here we review recent works examining how cancer cells become hypersialylated, how hypersialylation benefits cancer cells and tumors, and proposed therapies to abrogate hypersialylation of cancer.

## 1. A Growing Link between Hypersialylation, Cancer, and Inflammation

Inflammation is strongly implicated in playing key roles at all stages of cancer [1]. In earlier stages (transformation and angiogenesis) evidence supports inflammation as a driver of cancer progression [2]. In later stages, such as metastasis and in the tumor microenvironment, inflammation is exploited to mediate cancer cell invasion into secondary tissues and to shape immune responses in a way that favors tumor survival and progression, respectively. Tumors are particularly proficient at blunting immune cell responses directed at them by co-opting inhibitory receptors that keep the T-cells in an unresponsive state. With inhibitors of such immune checkpoints receiving much attention in recent years due to their ability to break the cycle of immune suppression and thereby enable immune cell killing of the cancer cells [3,4], there is heightened interest in examining additional mechanisms used by cancer cells to suppress and shape immune responses. One particular area of interest in regard to new immunotherapeutic potentials would be to target inflammation and, in particular, the immune cells responsible for inflammation, especially in certain forms of cancer where standard immune checkpoint inhibitors have minimal benefit [5].

**Figure 1:**
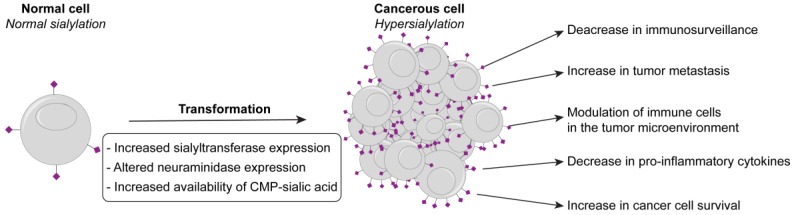
Hypersialylation in cancer: causes and effects. Elevated levels of sialic acid on transformed cells can be driven by at least three different mechanisms. Hypersialylation on cancer cells can promote tumor development and survival in a many of different ways but one key mechanism is through modulating immune cell responses and in particular those immune cells types involved in modulating the inflammatory environment in tumors.

One emerging mechanism under investigation as a potential new immune checkpoint is hypersialylation. Sialic acid is one of the key monosaccharide building blocks that composes cell surface glycans on mammalian cells. Sialic acid residues are strategically positioned at the tip of glycans, placing them at the forefront of many critical cellular processes involving cell–cell contact. Indeed, a growing body of evidence demonstrates that cancer cells have significantly elevated levels of sialic acid compared to non-transformed cells [6] (Figure 1). This has motivated investigation into the mechanisms behind how hypersiaylation enhances tumorogenesis through modulating immune cells [6,7,8]. Therapies are being proposed and tested in pre-clinical models that aim to decrease sialic acid on cancer cells or block key interactions between sialic acid and relevant receptors on myeloid cells that are crucial for maintaining the inflammatory environment in tumors [9,10,11,12]. This review highlights recent insights into mechanisms by which cancer cells become hypersialylated, evidence for how cancer cell hypersialylation alters immune cell responses to the cancer through modulating immune cells involved in inflammatory responses, and proposed therapies to break the cycle of hypersialylation and its effects. It is noteworthy that while clear lines can be drawn between hypersialylation and inflammation in some cases, in other cases the ways in which hypersialylation benefits cancer cells and tumors may not necessarily directly implicate inflammation. The goal of this review is to highlight the relevant mechanisms related to inflammation, but also briefly discuss mechanisms that are of general of interest in cancer.

## 2. Mechanisms Leading to Hypersialylation

For some time, tumor cells have been known to exhibit aberrant glycosylation, with increased levels of sialic acid being one of the more consistent and prominent changes across many different types of cancers [13]. Elevated levels of sialic acid can, in principle, arise through at least three different possible mechanisms (Figure 1). Here we will discuss these three possibilities, which are altered levels of sialyltransferases (ST) and neuraminidases (NEU), or altered substrate availability for the STs.

### 2.1. Sialyltransferase Expression

STs are a family of 20 enzymes that catalyze the linkage of sialic acid to the underlying glycan in four major linkages using cytosine monophosphate(CMP)-sialic acid as their donor substrate [14]. The regioselective addition of sialic acid onto the growing glycan structure by individual ST is crucial for recognition by glycan-binding proteins [15]. It has been shown that overexpression of at least 9 of the 20 STs is associated with malignant disease states (Table 1) [16]. Below we discuss the aberrant levels of STs that have been linked to cancer and their connection to inflammation where applicable. 

#### 2.1.1. ST6Gal1

ST6Gal1 adds sialic acid in an α2-6 linkage to an underlying galactose (Gal) residue. There are only two enzymes in humans that catalyze this particular linkage; ST6Gal1 being the primary enzyme, and ST6Gal2 having a restricted expression pattern [57]. ST6Gal1 is normally expressed in all homeostatic tissue but has been shown to be up-regulated in a variety of cancer tissues, including colon and breast, to name a few [17,28,58]. This up-regulation is likely due to the Ras oncogene and elevated levels of ST6Gal1 is correlated with a poor prognosis. Elevated ST6Gal1 expression makes cells resistant to TNF-α-induced apoptosis that is proposed to be mediated through addition of sialic acid to the TNFR1 death receptor [59]. An alternative mechanism by which overexpression of ST6Gal1 affects tumorgenesis is through blocking binding sites for Galectins, thereby inhibiting Galectin-dependent mechanisms such as induction of apoptosis [27,60] and modulation of immune cells [61]. Furthermore, it is thought that ST6Gal1 is involved in maintaining stem cell-like behavior within the cancer tissue, allowing transformed cells to evade multiple apoptosis pathways [17]. The product of ST6Gal1, α2-6-linked sialosides, have also been shown to modulate T-cell responses in the tumor microenvironment [28].

#### 2.1.2. ST3Gal1, ST3Gal3, ST3Gal4, and ST3Gal6

Six STs catalyze the transfer of sialic acid to an underlying Gal residue in an α2-3 linkage. ST3Gal1 acts predominantly on Core-1 O-glycans; these sialylated Core-1 O-glycans helps mask the tumor from surrounding immune cells [29]. More recently, sialylated Core-1 O-glycans on mucins were shown to promote tumorigenesis through engaging Siglec-9 on macrophages (see more below) [32]. Interestingly, it has been shown that overexpression of ST3Gal1 is induced by the up-regulation of cyclooxygenase-2, whose expression is significantly increased in times of inflammation [62]. Indeed, ST3Gal1 and cyclooxygenase-2 are upregulated in tumors and their expression levels correlate with metastatic disease potential that may be facilitated by decreased detection by immune cells [33,62]. The expression of ST3Gal3 has been also shown to be increased in a variety of cancers including pancreatic and gastric [18,34]. The overexpression of ST3Gal3 has been linked to cancer progression and is involved in creating ligands that can induce apoptosis in eosinophils [34,37]. Moreover, ST3Gal4 and ST3Gal6 are both involved with synthesizing sialyl-Lewis^x^ (and sialyl-Lewis^a^) and are upregulated in a many different cancer types [39,42,44,49,50]. Specifically, upregulation of ST3Gal4 and ST3Gal6 is linked to an increased metastatic and invasive disease state, as the glycan ligands they synthesize are able to bind Selectins that are well known to be expressed on inflamed vascular endothelial cells [41]. There is also evidence that glycans produced by ST3Gal4 and ST3Gal6 are also able to target regulatory T-cells to help suppress localized tissue inflammation [63].

#### 2.1.3. ST6GalNAc1 and ST6GalNAc2

ST6GalNAc1 is one of six STs that catalyze the transfer of sialic acid to an underlying N-galactosamine (GalNAc) residue in an α2-6 linkage. ST6GalNAc1 adds sialic acid to the Tn antigen (threonine with a single GalNAc). The resulting sialyl Tn-antigen (STn) is widely recognized as a glycan overexpressed in a variety of cancer types [64,65]. Overexpression of ST6GalNAc1 and elevated levels of STn have been shown to induce low levels of Th1-inducing cytokines, such as IL-12 and TNFα [47,51,66]. In the presence of human bladder cells that have high expression of STn, DC did not mature and produced less pro-inflammatory cytokines [47]. Furthermore, enhanced levels of the STn antigen can increase the invasiveness of cancer cells in a MUC1-dependent manner [67]. Conversely, the expression of ST6GalNAc2 is downregulated in malignant melanoma cells, and this downregulated expression positively correlates to malignant behavior [53,68]. Downregulated expression of ST6GalNAc2 causes a switch in the type of sialylated glycans present by increasing extended Core-2 O-glycans that coincides with an increase in Galectin-1 ligands [53]. Galectin-1 is found on a variety of immune cells, including inflammatory macrophages and natural killer (NK) cells, and is able to suppress immune responses through binding its cell surface glycan ligands [69,70,71].

#### 2.1.4. ST8Sia2 and ST8Sia4

ST8Sia2 and ST8Sia4 are two of six sialyltransferase enzymes capable of catalyzing the glycosidic linkage between two sialic acid residues in an α2-8 linkage. These two enzymes, in particular, are unique in this sub-family because they are capable of iteratively adding sialic acid to form polysialic acid [72]. Numerous studies have discovered that elevated levels of polysialic acid, due to overexpression of ST8Sia2 and ST8Sia4, on cancer cells is important in the etiology and pathophysiology of a number of cancer types. ST8Sia2 is not expressed at significant levels in most adult tissues, as its expression is tightly regulated postnatally [73]. On the other hand, ST8Sia4 is expressed in adults but is restricted primarily to the brain [74]. The expression of ST8Sia2 and ST8Sia4 has been shown to be elevated in the tumors of patients with astrocytoma, which correlates with an increase in polysialic acid that is linked to tumor invasiveness [54]. The increase in ST8Sia2 expression has also been correlated to increased phosphorylation levels of ERK1/2, MMP-9, and FGFR1, all leading to increased invasion and migration of cells in vitro [75]. On the other hand, the overexpression of ST8Sia4 has been shown to be positively correlated to multi-drug resistance in chronic myeloid leukemia by regulating the PI3K/Akt signaling pathway, leading to an increase in cell survival [55]. ST8Sia4 has also been found to be expressed at higher levels than normal tissue in the breast cancer cell line MDA-MB-231, where the increased cellular proliferation and invasiveness associated with this metastatic cell line are significantly decreased upon knockdown of ST8Sia4 [56].

### 2.2. Increased Availability of CMP-Sialic Acid

The substrate availability of STs, in the form of levels of their donor (CMP-sialic acid) or acceptor (underlying glycoconjugate) have the potential to affect levels of cell surface sialic acid. Intracellular increases of sialic acid can occur through different pathways, one of which being metabolic flux-driven changes, where changes in the hexosamine biosynthetic pathway can alter the sialic acid metabolic pathway through varying the levels of UDP-GlcNAc [76]. It is noteworthy that increased levels of UDP-GlcNAc can qualitatively and quantitatively alter N-glycosylation patterns through increased branching that provides more acceptor sites for sialylation [77]. Increases in the UDP-GlcNAc levels can also lead to an increase in transcription of sialyltransferases and the addition of sialic acid to proteins, such as MUC1, is associated with metastasis in cancer [78]. There is also evidence that mammary cells can switch to the hexosamine metabolism pathway when stimulated by oncogenic stimuli in order to increase the amount of CMP-sialic acid and sialylated glycoconjugates [79]. Interestingly, a recent study demonstrated that cancer cells regulate intracellular levels of sialic acid in a way that is dependent on the stage of the cancer [80]. Overall, due to challenges in measuring intracellular levels of CMP-sialic acid, studies examining how CMP-sialic acid levels change in cancer is warranted.

### 2.3. Neuraminidases

There are four mammalian neuraminidases (NEU1–4), which cleave sialic acid residues from cellular glycoconjugates. Individual neuraminidases have been linked to certain cancer types. For example, NEU1 has been linked to cancer metastasis, as it is involved in facilitating the epithelial to mesenchymal transition in cancer cells [81]. NEU2 has the most dramatic results with regard to cancer; one study has shown that when NEU2 is overexpressed there is increased cell survival in prostate cancer [82]. Controversially, NEU2 expression has been shown to increase tumor cell apoptosis in a leukemia cell line [83]. NEU3 has been shown to be overexpressed in a large number of cancer types including prostate cancer [84], melanoma [85,86], and ovarian cancer [87]. NEU3 is able to remove sialic acid from the gangliosides GM3 and GD1a, generating ligands for the EGFR receptor and activate the ERK1/2 pathway [88]. Overexpression of NEU3 is expected to cause hyposialylation, but gangliosides are overexpressed in different cancer types, such as breast and melanoma [89,90]. Tumors can use this overexpression of NEU to release membrane fragments with GM3 and GD1a, allowing for protection from immune cells and correlating with tumor invasiveness and metastases [91,92,93]. On the other hand, NEU4 is down-regulated in cancers, allowing for the increased expression of sialyl-Lewis^x^ (and sialyl-Lewis^a^) carbohydrate structures, leading to increased tumor metastasis [94]. However, NEU4 expression and its link to cancer and inflammation are not well understood at this point.

## 3. How Altered Sialic Acids Modulate Inflammatory Responses by Immune Cells

Numerous mechanisms have been forwarded for how hypersialylation modulates host immune response to cancer cells. Here, we focus on how this modulation relates to controlling or exploiting inflammatory responses, such as modulation of immune cells by Siglecs, roles for Selectins in mediating cancer metastasis, and antibodies directed at non-human type sialic acid. It is important to note that numerous other mechanisms have been proposed for how hypersialylation benefits tumorogensis, including: avoidance of complement binding through Factor H recognition of sialic acid [95,96], direct skewing of NK and T-cell responses [97,98], and intrinsic functions imparted by sialic acid to the cancer cells [99,100].

### 3.1. Siglecs

**Figure 2:**
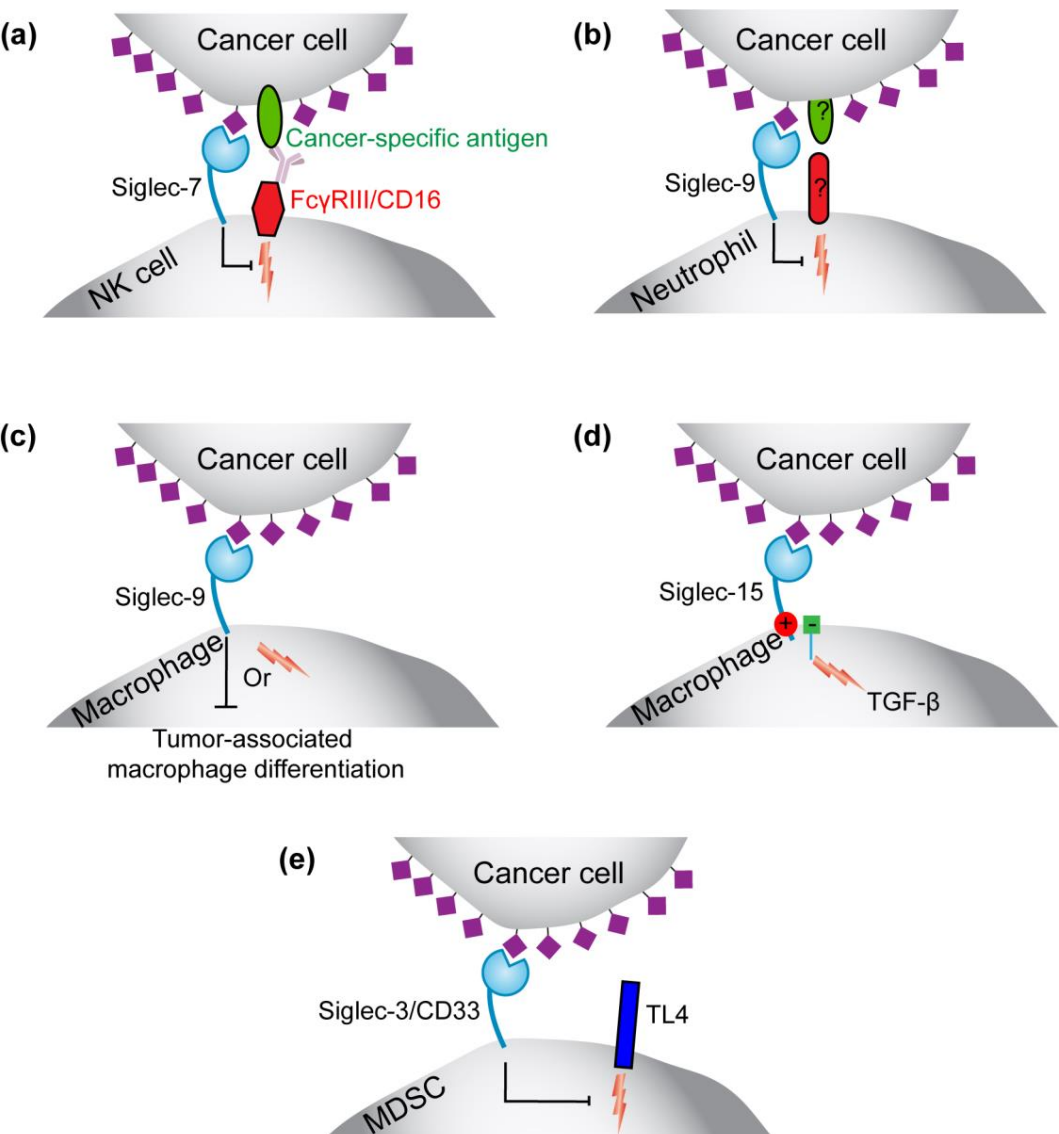
Roles for hypersialylation on cancer in modulating Siglecs on immune cells. (**a**) Siglec-7 (and potentially Siglec-9) on NK cells can be drawn into an immunological synapse formed with a cancer cell to inhibit an activatory receptor (e.g., CD16), thereby inhibiting NK cell-mediated killing; (**b**) Siglec-9 on neutrophils can be engaged by hypersialylation of cancer cells and prevent neutrophil-mediated killing of cancer cells through an unknown activatory receptor and ligand on the cancer cells; (**c**) Siglec-9 on macrophages can promote or inhibit skewing of macrophages to a tumor-associated or tumor-promoting phenotype through engagement of sialic acid-containing glycans on cancer cells; (**d**) Siglec-15 on macrophages pairs with the adapter protein Dap12 to activate cellular signaling in response to sialic acid ligands on cancer cells, thereby inducing TGF-β production; (**e**) CD33 (Siglec-3) on myeloid-derived suppressor cells (MDSCs) has been shown to modulate inflammatory responses through modulating TLR4.

Sialic acid-binding immunoglobulin-type lectins (Siglecs) are cell surface receptors that bind sialic acid-containing glycoconjugates [15]. In humans, 15 Siglec family members are differentially expressed on white blood cells, although it should be noted that there is a growing list of examples for Siglecs expressed outside of the immune cell lineage [101,102,103,104]. Siglecs can modulate immune cell signaling in both positive or negative ways. Many of the Siglecs are inhibitory by virtue of the fact that they contain immunotyrosine-based inhibitory motifs (ITIMs) on their cytoplasmic tail. This inhibitory motif can be phosphorylated under the appropriate physiological or pathophysiological circumstance, resulting in recruitment of SH2-containing phosphatases, which thereby dampen immune cell signaling. On the other hand, Siglecs 14–16 do not contain an ITIM but, instead, contain a lysine residue in their transmembrane region that facilitates pairing with immunotyrosine-based activatory motif (ITAM)-containing adaptor proteins, such as DAP12 [105]. Recently, the lines between inhibitory and activatory Siglecs have been somewhat blurred, where inhibitory Siglecs have been shown to induce cellular signaling [32,106,107]. In any case, the ability of Siglecs to recognize sialic acid, which is upregulated on cancer, coupled with altering immune cell responses, such as modulation of inflammatory responses, is a growing area of investigation with numerous proposed mechanisms (Figure 2) [8,108].

#### 3.1.1. Roles for Siglec-7 and -9 on NK Cells and Siglec-9 on Neutrophils in Immunosurveillance

Natural killer (NK) cells and neutrophils are innate immune cells capable of responding fast and killing virus-infected or transformed cells. Human NK cells highly express Siglec-7 with a subset also expressing Siglec-9. As originally proposed by Crocker and co-workers, cancer cells upregulate expression of sialic acid-containing Siglec-7 ligands to engage Siglec-7 on NK cells, resulting in inhibition NK-mediate killing of cancer cells [109] (Figure 2a). More recently, this was shown more directly and globally on various cancer cells lines [63] and demonstrated that hypersialylation of cancer cells indeed inhibits the response of NK cells toward cancer cells in a predominantly Siglec-7 dependent manner [110,111]. To fully investigate the in vivo consequence of these interactions and the therapeutic potential of breaking such interactions will require the development of a human Siglec-7 transgenic mouse, which is particularly relevant given that murine NK do not endogenously express any Siglecs at high levels. Neutrophils are also capable of responding quickly and are implicated in the immune surveillance of cancer, but clearly have the potential to be both pro- or anti-cancer depending on the circumstances [112]. Expression of Siglec-9 or its murine counterpart Siglec-E on neutrophils was shown to dampen the response of neutrophils to cancer cells (Figure 2b), as highlighted by the fact that more cancer cells were able to colonize the lung in short-term assays in mice expressing neither Siglec-E nor Siglec-9 [113]. This exploitation of inhibitory Siglecs by cancer cells overexpressing sialic acid on their cell surface is a similar mechanism as described above for Siglec-7 on NK cells. Consistent with the dampening of responses of immune cells to cancer cells by Siglec-9, polymorphisms in Siglec-9 that partially abrogate sialic acid binding gives rise to moderately better early survival in patients with non-small cell lung carcinoma (NSCLC) [113]. One unexplored area is the role that Siglecs play on tumor-associated neutrophils, that are thought to benefit tumors by promoting inflammation and recruiting tumor-associated macrophages.

#### 3.1.2. Siglec-9 in Regulating the Function or Formation of Tumor-Associated Macrophages

Siglec-9 has been investigated in detail in two prominent studies looking at its role on macrophages in the context of cancer. What has emerged are two models. In the first study by Laubli et al., the authors found that expression of Siglec-9, or its murine counterpart Siglec-E, slowed down tumor growth within an established murine tumor model [113]. Specifically, the model proposed that engagement of Siglec-9 with its sialic acid-containing ligands on the cancer cells inhibited the skewing of macrophages to M2 polarized tumor-promoting macrophages (Figure 2c). The evidence for this model was primarily based on the fact that tumors implanted into mice expressing neither Siglec-9 or Siglec-E, once established, grew faster, which was suggested to be due to an abundance of tumor-associated macrophages (TAMs) that aided tumor growth. In the second study, by Beatson et al., an alternative model was revealed [32]. Working entirely in vitro, the authors of this study found that engagement of Siglec-9 by tumor-produced mucins, in particular MUC1, enhanced skewing of macrophages to a more TAM-like phenotype (Figure 2c). Curiously, MUC1 engagement of Siglec-9 was shown to drive cellular signaling, which is counterintuitive to how inhibitory Siglecs normally function. Unfortunately, the consequence of these effects in the tumor microenvironment was not tested. Reconciling the two somewhat opposing models presented in these two studies is rather difficult, but could relate to the different types of cancer investigated or the fact that in the first study, expression of Siglec-E and -9 were not entirely confined to the macrophages lineage, meaning that Siglec-dependent effects on other immune cell types could have been at play. Regardless, these results clearly motivate further testing of these hypotheses.

#### 3.1.3. Siglec-15 on Tumor-Associated Macrophages

Siglec-15 is a member of the Siglec family that pairs with the adapter protein Dap12. Expression of Siglec-15 on TAMs has been observed [114,115]. In an in vitro model system using co-culture of lung carcinoma cells with a Siglec-15 expressing monocyte/macrophage cells, it was demonstrated that engagement of sialic acid ligands from the cancer cells with Siglec-15 on the macrophages enhanced TGF-β production (Figure 2d). The fuller significance of these results awaits in vivo studies but the link between Siglec-15 and cancer is intriguing given that Siglec-15 is a non-conical member of the Siglec family capable of positively regulating immune cell signaling.

#### 3.1.4. Siglec-3 (CD33) on Myeloid-Derived Suppressor Cells (MDSCs)

Myeloid-Derived Suppressor Cells (MDSCs) are a type of innate white blood cell present in healthy people at fleeting numbers, but is strongly up-regulated in cancer patients with particular concentrations within the tumor. Many studies have used CD33 as a marker of MDSCs, although markers to distinguish MDSCs from other myeloid cells (neutrophils and monocytes) are still emerging [116]. In one study, it was found that CD33 modulates the suppressive properties (e.g., IL-10 production) of MDSCs, which was suggested to take place through abrogating TLR4 signaling [117] (Figure 2e).

### 3.2. Selectins

Selectins are a family of C-type lectins that recognize their glycoconjugates (sialyl-Lewis^x^-containing glycoproteins) in a sialic acid-dependent manner [118]. E- and P-Selectin are expressed on inflamed endothelial cells where they play a role in the process of leukocyte rolling and adhesion [119]. Nevertheless, through overexpressing sialic acid-containing Selectin ligands, cancer cells are thought to exploit this inflammatory mechanism by promoting metastasis through extravasation [10,120,121,122]. It has been shown that overexpression of ST6Gal6 in multiple myeloma created Selectin ligands, contributing to enhancing homing and survival of the cancer cells in vivo [42]. A similar phenomenon has also been observed for overexpression of ST3Gal3 and ST3Gal4 in highly metastatic colon cancer, whereby they create high levels of E-Selectin ligands [123]. More recent studies have begun to shed light on more diverse roles for Selectin-glycan interactions in cancer cells, such as upregulation of P-selectin to mediate adherence to cancer cells [124], induction of cellular signaling in the cancer cells [125], and cancer-cell induced activation of platelets that induces trapping of cancer cells in the lung [126].

### 3.3. Neu5Gc

*N*-glycolylneuraminic acid (Neu5Gc) is one of the major forms of sialic acid in most mammals, but it cannot be biosynthesized in humans due to the loss of the CMP-sialic acid hydroxylase (CMAH) enzyme [127,128,129]. Despite the fact that Neu5Gc cannot be produced biosynthetically in human cells, it can be incorporated into glycans by scavenging Neu5Gc from dietary sources [130]. Indeed, following intracellular hydrolysis of Neu5Gc from exogenous glycoconjugates, it can then be converted to CMP-Neu5Gc and used as a substrate by the STs [130]. Anti-Neu5Gc antibodies have been shown to be produced in humans in responses to this foreign form of sialic acid and these xenoautoantibodies have been linked to inflammation and tumorigenesis. Specifically, it was demonstrated that in ‘humanized’ mice lacking the CMAH gene, anti-Neu5Gc antibodies developed after vaccination with Neu5Gc-containing glycoconjugates and that upon feeding Neu5Gc rich glycoproteins, there was an increased rate of liver cancer [127]. The proposed mechanism for the increase cancer occurrence is through the xenoautoantibodies that drives inflammation and particularly the pro-inflammatory cytokine IL-6 [127].

## 4. Potential Treatment Strategies

**Figure 3:**
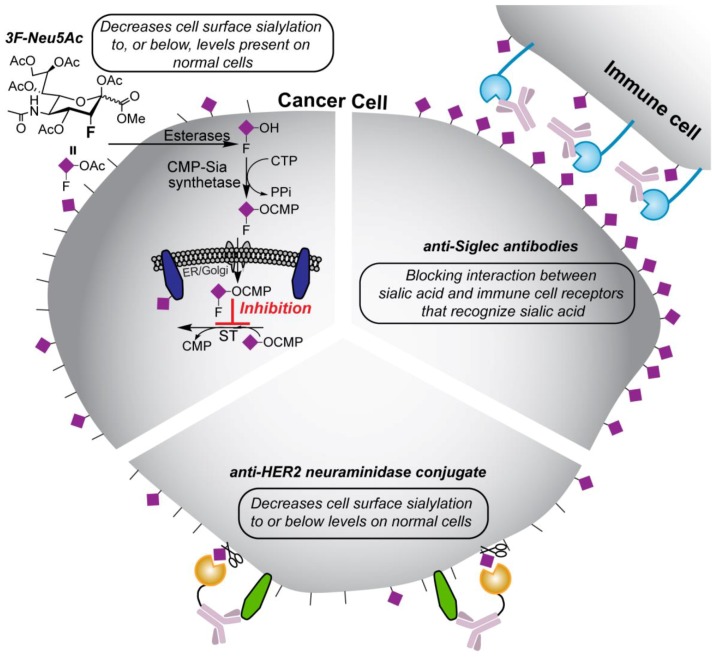
Therapeutic strategies for modulating sialic acid expression and interactions between sialic acid and sialic acid-binding receptors. (**Upper left portion of cell**): delivery of 3F-Neu5Ac to cancer cells to decrease sialic acid expression. 3F-Neu5Ac is typically delivered in its peracetylated form to penetrate the cell membrane. Once inside the cell, 3F-Neu5Ac is converted into CMP-3F-Neu5Ac and blocks the actions of STs in the secretory pathway. (**Upper right portion of cell)**: blocking antibodies that abrogate the interaction between sialic acid on cancer cells and sialic acid-binding receptors on immune cells, such as Siglecs, have the potential of being a new class of immune checkpoint inhibitor. (**Lower portion of the cell**): antibody directed to the tumor, such as anti-HER2 to target breast cancer cells, have been conjugated to neuraminidase to cleave the sialic acid residues on the cancer cells.

With the recent success of immune checkpoint inhibitors, there is a keen interest to establish whether breaking other inhibitory circuits, particularly those in innate immune cells that perpetuate inflammation within the tumor, can also produce therapeutic results. As described above, a growing body of evidence suggests that sialic acid on cancer cells modulates immune responses in the tumor microenvironment, and strategies to break these interactions are starting to emerge (Figure 3). These approaches are also serving to reinforce the causal link between hypersialylation and tumorigenesis.

### 4.1. Targeting Neuraminidase to Cancer Cells

Neuraminidase treatment of a variety of cancer cells removes Siglec-7 and Siglec-9 ligands on cancer cell and promote cancer cell killing by NK cells in vitro as well [110]. In parallel, it was also demonstrated that neuraminidase-treated cancer cells injected in mice with human NK cells were cleared faster [111]. These examples highlight the opportunity to aid NK cells in killing cancer cells in the early stages of immunosurveillance. To take this concept to the next level, Xiao et al. developed a neuraminidase that is targeted to cancer cells by conjugation to a tumor-targeting antibody [131] (Figure 3; lower part of cell). As shown for the anti-HER2 antibody, neuraminidase conjugation enabled anti-HER2 antibody to selectively target breast cancer cells in vitro and increase the effectiveness of NK cell killing of these cells. It is intriguing to think about how this approach could also bring about additional benefits in vivo by abrogating other aspects through which sialic acid is involved in modulating immune cells in the tumor. Recently, it has also been demonstrated that targeting an endo-neuraminidase to cancer has therapeutic potential [132]. In this work, endo-neuraminidase was fused to oncolytic adenovirus for delivery to the tumor and this approach was demonstrated to not only target the virus to the tumor, but also resulted in degradation of the polysialic acid on the cancer cells.

### 4.2. Anti-Siglec and Anti-Selectin Antibodies

Anti-Siglec antibodies may also be a way of blocking Siglec-ligand interaction or modulating immune cell function (Figure 3; upper right portion of cell). Ligand-blocking antibodies were shown to prevent the inhibition that Siglec-9 had on TAMs [113]. Likewise, anti-Siglec-7 antibodies were effective in blocking interaction between the two respective Siglecs and their sialic acid-containing glycans on cancer cells to potentiate NK cell killing [110]. Careful inspection of the data using the only commercially available anti-Siglec-9 ligand-blocking antibody, however, suggests that this antibody may itself activate myeloid cells expressing Siglec-9 [32,113,133]. Whether blocking endogenous ligand interactions between Siglec-9 and its ligands on the same cell surface drives cell signaling is not entirely clear but warrants careful investigation in future studies. In terms of engaging Siglec-9, it is intriguing to note that nanoparticles bearing ligands of Siglec-9, or its functional counterpart in mice Siglec-E, produces tolerogenic effects in human and mouse macrophages, respectively [134]. Likewise, antibody to Siglec-9 showed anti-inflammatory effects macrophages [135]. Therefore, while anti-Siglec-9 antibodies hold promise as a new immunotherapy, what effect antagonistic or ligand-blocking antibody produce in the tumor microenvironment remains to be determined. Anti-Selectin antibodies, antibodies that recognize selectin ligands, or other platforms to block Selectin-mediated recognition of their endogenous ligands have been contemplated as a means of blocking cancer metastasis [10,136,137]. It is noteworthy that other pharmacological approaches to disrupting Selectin-ligand interactions are also being pursued [125,138,139,140,141].

### 4.3. Sialyltransferase Inhibition

In order to block incorporation of sialic acid onto cell surface glycans, several targets are plausible. For example, based upon work using knockdown of the CMP-sialic acid transporter [97], which is responsible for transport of CMP-sialic acid into the Golgi, this was identified as a potential druggable target. Indeed, earlier efforts were made to diminish the intracellular pools of CMP-sialic acid as a way of decreasing ST activity through the development of an inhibitor of the CMP-sialic acid transporter [142]. This inhibitor, identified many years ago, was shown to decrease levels of cell surface sialic acid and inhibit the formation of metastases in human colorectal cancer cells within a mouse model. Inhibition of the STs is also a viable route to diminishing levels of cell surface sialic acid; this approach for diminishing sialic acid content in cancer cells has been pursued (Figure 3; upper left portion of cell). There is only one known inhibitor of STs that works in cells, which is a global ST inhibitor when used in its peracetylated form for cell permeability [143]. This inhibitor, termed 3F-Neu5Ac, diminishes sialic acid content in cells by approximately 80–90% through the actions of being converted into CMP-3F-Neu5Ac and failing to be used as a substrate by the STs. However, due to the multi-facetted physiological roles of sialic acid, it was revealed that while 3F-Neu5Ac is extremely effective in vivo, it produces kidney dysfunction due to specific removal of sialic acid residues from podocytes, thus destroying glomerular filtration in the kidney [144]. Recognizing this limitation, Adema, Boltje, and co-workers have investigated several methods for selectively delivering 3F-Neu5Ac to the tumor. In the first approach, cancer cells were simply fed 3F-Neu5Ac in vitro prior to being implanted into mice, which resulted in decreased engraftment of cancer cells as well as tumor growth [145]. In the second approach, nanoparticles encapsulated with 3F-Neu5Ac bearing anti-tyrosinase-related protein-1 antibody were used to direct the compound to the tumor [146]. This targeted delivered strategy resulted in decreased sialic acid levels on the tumor cells as well as a decrease in the number of lung nodules. More recently, the global ST inhibitor was injected directly into tumors and remarkable changes in the T-cell subsets were found, including an increase in effector CD4^+^ and CD8^+^ T-cells that were mirrored by a decreased in regulatory T-cells [98]. Despite the localized site of injection, the authors did note renal toxicity at higher doses, pointing to the need for better tolerated versions of 3F-Neu5Ac for in vivo use.

## 5. Conclusions

Studies examining sialylated glycans as immune checkpoints are in their infancy but advancing at a rapid pace. Due to the differences between Siglecs in mouse and humans, development in mouse models expressing human Siglecs on relevant immune cells [113,147,148,149], or mouse models engrafted with human immune cells that have been genome-edited to modulate Siglec expression [150], should greatly accelerate our understanding of the role Siglecs play in the tumor microenvironment and enable the testing of therapies aimed at abrogating sialic acid-Siglec interactions.

## Figures and Tables

**Table 1:** Altered sialyltransferase expression in cancer and its relation to inflammation.

Sialyltransferase	Cancer-Specific Glycosylation Change	Cancer Types	Gene Expression Changes	Link to Inflammation
ST6Gal1	Increase in α2-6 sialosides	Epithelial [17], gastric [18], leukemia [19], breast [20], colorectal [21], acute myeloid leukemia [22], choriocarcinoma [23], cervix [24], brain [25], liver [26]	Elevated	Evading TNFα induced apoptosis [27]; Promoting naïve CD4^+^ T cell differentiation into regulatory T-cells [28]
ST3Gal1	Increased α2-3 sialylated Core 1 O-glycans	Breast [29], bladder [30], colon [31]	Elevated	Modulated macrophage differentiation [32]; Masking of tumor from surrounding immune cells [33]
ST3Gal3	Increased sialyl-Lewis^a^, sialyl-Lewis^a^	Pancreas [34], gastric [18], bile duct [35], cervix [36]	Elevated	Induction of apoptosis on eosinophils [37]
ST3Gal4	Increased sialyl-Lewis^a^, sialyl-Lewis^a^	Gastric [38], renal [39]	Elevated	Targeting of regulatory T-cells to suppress tissue-localized inflammation [40]; Promoting metastasis through creating Selectin ligands [41]
ST3Gal6	Increased sialyl-Lewis^a^, sialyl-Lewis^a^	Multiple myeloma [42], liver [43], gastrointestinal [44], adenocarcinoma [45], breast [46]	Elevated	
ST6GalNAc1	Increase sialyl Tn	Breast [47], colon [48], adenocarcinoma [49], gastric [50]	Elevated	Induction of low levels of Th1-inducing cytokines [47,51]; Blocking of dendritic cell (DC) maturation [47]
ST6GalNAc2	Increased extended Core 2 O-glycans	Colerectal [52], melanoma [53]	Decreased	Increase in Galectin-1 ligands to suppress immune response [53]
ST8Sia2	Increased polysialic acid	Liver [26], astrocytoma [54]	Elevated	Modulation of PI3K/Akt pathway to negatively regulate pro-inflammatory cytokines [55]
ST8Sia4	Increased polysialic acid	Chronic myeloid leukemia [55], breast [56], astrocytoma [54]	Elevated	

## Abbreviations

NSCLC	non-small cell lung carcinoma
Siglec	sialic acid-binding immunoglobulin-type lectin
NK	natural kill
Neu5Ac	*N*-acetylneuraminic acid
Neu5Gc	*N*-glycolylneuraminic acid
CMAH	CMP sialic acid hydroxylase
CMP	cytosine monophosphate
UDP	uridine diphosphate
GlcNAc	*N*-acetylglucosamine
ST	sialyltransferase
ITIM	immunotyrosine-based inhibitory motif
ITAM	immunotyrosine-based activatory motif
Neu	neuraminidase
MDSCs	myeloid-derived suppressor cells
Gal	galactose
GalNAc	*N*-galactosamine
STn	sialyl Tn antigen
